# Analyzing the effectivity of evidence-based practice in health science higher education: a narrative review

**DOI:** 10.1590/1516-3180.2023.0407.R1.05062024

**Published:** 2025-01-27

**Authors:** Victória Guzzo da Silva, Dafne Port Nascimento, Sarah Beatriz Obadovski Alves Nascimento, Amanda Costa Araujo

**Affiliations:** I Master’s Program in Innovation in Higher Education in Health, Universidade Municipal de São Caetano do Sul (USCS), São Caetano do Sul (SP), Brazil.; II Masters and Doctoral Programs in Physical Therapy, Universidade Cidade de São Paulo (UNICID), São Paulo (SP), Brazil.; III Professor, Centro Universitário Integrado, Campo Mourão (PR), Brazil.; IV Professor, Master’s Program in Innovation in Higher Education in Health, Universidade Municipal de São Caetano do Sul (USCS), São Caetano do Sul (SP), Brazil.

**Keywords:** Education., Health promotion., Evidence-based practice., Learning., Teaching., Health Education., Health sciences., Higher education.

## Abstract

**BACKGROUND:**

Although multiple strategies have been suggested for evidence-based practice educational interventions, few studies have focused on the development of abilities for evidence-based practice implementation.

**OBJECTIVE:**

To explore the effectiveness of evidence-based practice in higher education and understand its teaching methods.

**DESIGN AND SETTING:**

Narrative review was conducted at the Universidade Municipal de São Caetano do Sul, Brazil.

**METHODS:**

Narrative review included research studies that measured any type of evidence-based practice teaching method and its effectiveness. Searches included publications from inception to June 2022, conducted on MEDLINE, EMBASE, CINAHL, CENTRAL, ERIC, and the Cochrane Library. Two independent authors descriptively extracted and analyzed the data. The methodological quality of the studies was also analyzed.

**RESULTS:**

The results determined that 79.2% of the studies proved their effectiveness. Teaching methods varied according to the time period, format, and types of questionnaire.

**CONCLUSIONS:**

Most studies demonstrated the effectiveness of the chosen teaching methods. This study shows the importance of health professionals using evidence-based practice to ensure effective patient treatment.

## INTRODUCTION

Evidence-based practice (EBP) consists of the best available scientific evidence, previous professional clinical experience, and patients’ preferences.^1^ EBP use seeks to improve the efficiency and quality of healthcare services, besides saving costs and expenses with inefficient treatments.^2^ In recent years, EBP implementation has proven great advances in improving healthcare quality and prognosis of various health conditions.^1^

However, some obstacles may interfere with EBP, such as limited resources, the ability to apply adequate intervention, cultural and socioeconomic factors, issues related to valid health policies, clinical practice complexity, access to full texts of scientific articles, and continuing education programs.^3,4^ In addition to those barriers, it is evident that during the entire learning trajectory, students are not taught and stimulated to have critical appraisal.^5^ Many times, students assume a passive posture in the learning process and end up losing the possibility to practice and absorb didactical content without depending on a tutor to graduate.^5^

In the teaching context for healthcare professionals graduation courses, EBP must follow five basic steps to reach success on its principle application: 1) formulation of clinical question; 2) broad and efficient search in health databases; 3) critical appraisal of evidence validity; 4) application of evidence results in clinical practice; and 5) assessment of treatment effects in your own clinical practice.^6^ Higher education courses have been seeking the most efficient approach to teach the necessary abilities of EBP; therefore, students turn into confident professionals when taking their clinical decisions.^7^ The most efficient approach is quite challenging,^8,9^ as one of the greater challenges is to adequate the theoretical structure to support and develop EBP requirements.^7^

Furthermore, a systematic review^10^ of 20 articles reporting the application of EBP educational interventions in healthcare suggested that multiple strategies, such as technology and/or simulation techniques, may influence the abilities, knowledge, and attitudes regarding EBP use. The results describe strategies focused mainly on teaching critical appraisal of information; however, only a few studies have focused on the development of abilities for EBP implementation.

## OBJECTIVE

Therefore, the primary objective of this study is to explore the effectiveness of EBP in higher education. The secondary objective is to understand the methods used to teach EPB in higher education.

## METHODS

### Inclusion and exclusion criteria

This narrative review included all original research studies that measured any type of EBP teaching method and their effectiveness. The question based on Population, Intervention, Comparison, and Outcome was as follows: Is EBP effective in higher education? Letters to the editor, editorials, and conference abstracts were excluded. Studies that included health professionals as the study population rather than students were also excluded.

### Search strategy for identification of studies

We searched publications published from the inception to June 9, 2022, with no language restrictions. We conducted our search on MEDLINE, EMBASE, CINAHL, CENTRAL, ERIC, and Cochrane Library, with two key search terms: “evidence-based practice” and “graduate education” or “higher education” (**Table 1**).

**Table 1:** Details of the search strategy

Database	Search strategies	Papers found
Medline (via Pubmed)	(“evidence-based practice”) AND (“graduate education”) OR (“higher education”)	2,974
ERIC	(“evidence-based practice”) AND (“graduate education”) OR (“higher education”)	1,000
CINAHL	(“evidence-based practice”) AND (“graduate education”) OR (“higher education”)	306
CENTRAL	(“evidence-based practice”) AND (“graduate education”) OR (“higher education”)	70
EMBASE	(“evidence-based practice”) AND (“graduate education”) OR (“higher education”)	26
Cochrane Library	(“evidence-based practice”) AND (“graduate education”) OR (“higher education”)	0

Medline = Medical Literature Analysis and Retrieval System Online; ERIC = Education Resources Information Center; CINAHL = Cumulative Index to Nursing and Allied Health Literature; CENTRAL = Cochrane Central Register of Controlled Trials; EMBASE = Excerpta Medica Database.

### Data collection

Two independent authors (PR and VS) screened all studies for eligibility. Disagreements were resolved through discussion or arbitration with a third author (AA). The screening process of the studies included (1) screening titles and abstracts and (2) screening full-text articles.

### Data extraction

Two independent authors (AP and KO) extracted the following data: (1) first author, (2) year of publication, (3) research field, (4) study objectives, (5) year of search, (6) sample size, (7) EBP teaching method, (8) how data were extracted, and (9) conclusions based on the study results regarding the effectiveness of the chosen method. If required, we contacted the authors via email to request any information that was not reported in the original manuscript.

### Data analysis and methodological quality of studies

The results are reported descriptively. Two independent authors (AA and VS) assessed the methodological quality of the studies using the Risk of Bias In Non-randomized Studies of Interventions (ROBINS-I) tool,^11^ which was developed to assess the methodological quality of quasi- or non-randomized experimental studies using seven items. Each item was rated as “Yes,” “Probably yes,” “Probably no,” “No,” or “No information” and the categories for risk of bias judgments were “Low risk,” “Moderate risk,” “Serious risk,” and “Critical risk” of bias. Both authors rated the risk of bias of studies on the number of “Yes” items, none as “Low,” one or two as “Moderate,” three as “Serious,” and more than three as “Critical.” During judgment, authors considered items rated “Probably yes,” “Probably no,” and “No information” as “Yes.”^11^

### Ethics and registration

Ethics approval was not required for this study. This review has not been registered as it does not have any health-related outcomes.^12^

## RESULTS

### Search results

**Figure 1:**
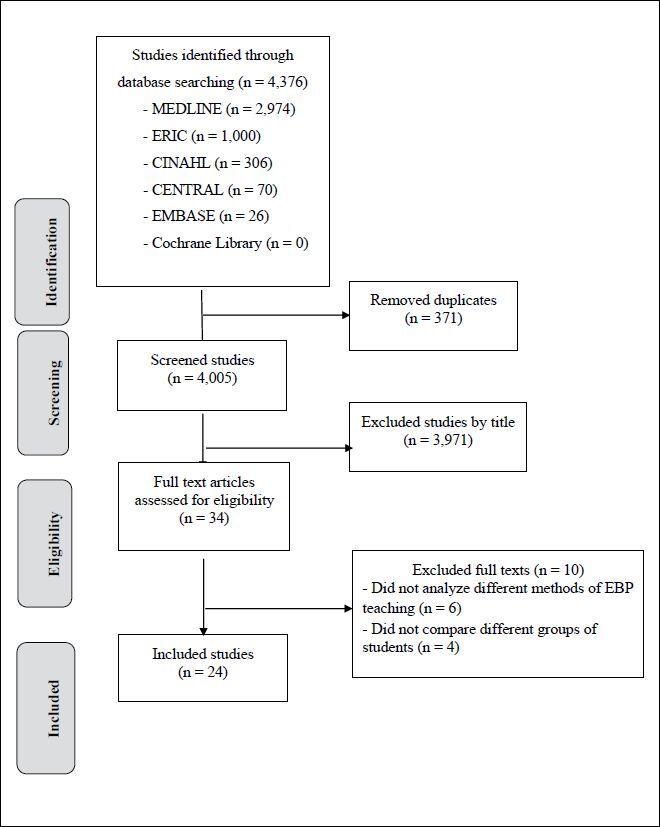
Review flow diagram.

The initial search yielded 4,376 potentially eligible studies. After screening the title and abstract, we removed duplicates, considered 34 potentially eligible studies for inclusion, and retrieved the full-text articles. Twenty-four published studies^13,14,15,16,17,18,19,20,21,22,23,24,25,26,27,28,29,30,31,32,33,34,35,36^ met the inclusion criteria and were included in this review. A review flow diagram is presented in **Figure 1**.

### Methodological quality of studies

The design of most of the studies was quasi-experimental, which is why the authors chose the ROBINS-I tool for methodological quality assessment. Most studies presented a moderate risk of bias (n = 14; 58.3%), followed by low (n = 4; 16.7%), serious, and critical (n = 3; 12.5% each). The methodological quality of each study is demonstrated in **Table 2**.

**Table 2:** Methodological quality assessment of the included studies, using the ROBINS-I tool^11^

Author, year of publication	Pre-Intervention		At Intervention	Post-Intervention				Risk of bias
Arias, Peters, Broyles, 2017^13^	Y	N	Y	Y	N	Y	Y	Critical
Bennett, Hoffmann, & Arkins, 2011^14^	Y	Y	N	N	N	N	N	Moderate
Durieux, Maillart, Donneau, Pasleau, 2018^15^	N	PN	N	N	Y	N	N	Moderate
Feldstein, Maenner, Srisurichan, Roach, Vogelman, 2010^16^	N	N	N	N	N	N	N	Low
George, Reis, Nothnagle, 2012^17^	N	N	N	N	N	N	Y	Moderate
Green Ellis, 1997^18^	N	N	N	N	N	N	N	Low
Hadley et al., 2010^19^	N	PN	N	Y	Y	N	PN	Serious
Harewood Hendrick, 2010^20^	N	N	N	N	N	N	N	Low
Jackson, 2016^21^	N	N	N	Y	Y	N	Y	Serious
Kenefick, Boykan, Chitkara, 2013^22^	N	N	N	N	N	Y	Y	Moderate
Kortekaas et al., 2016^23^	N	N	N	N	Y	N	N	Moderate
Ma, Chang, Krupat, 2021^24^	N	N	N	PY	Y	N	N	Moderate
McCluskey Lovarini, 2005^25^	N	N	N	N	N	N	N	Low
Mlika, Ben Hassine, Charfi, Mezni, Jouini, 2019^26^	N	N	N	N	N	Y	Y	Moderate
Moore, Watters, Wallston, 2019^27^	N	N	N	N	Y	Y	Y	Serious
Perraton et al., 2017^28^	N	N	N	N	N	N	Y	Moderate
Schweikhard, Hoberecht, Peterson, Randall, 2018^29^	N	N	N	N	N	N	PY	Moderate
Soma, Homme, Jacobson, 2013^30^	N	N	N	N	N	N	Y	Moderate
Tavarez, Kenkre, Zuckerbraun, 2020^31^	N	N	N	N	N	N	PY	Moderate
Thom, Haugen, Sommers, Lovett, 2004^32^	N	N	N	Y	PY	Y	Y	Critical
Thomas et al., 2005^33^	Y	Y	Y	N	N	Y	Y	Critical
Wadgave, Khairnar, Kadu, Chadha, Wadgave, 2020^34^	N	N	N	N	N	N	PN	Moderate
Wang et al., 2017^35^	N	N	N	N	NI	N	Y	Moderate
Welch, Van Lunen, Hankemeier, 2014^36^	N	N	N	Y	N	Y	N	Moderate

Y = yes; N = no; PY = probably yes; PN = probably no; NI = no information.

### Characteristics of included studies

The 24 eligible studies were published between 1997 and 2019. Study designs varied among included studies: 16 quasi-experimental studies,^13,14,15,17,18,21,22,24,25,27,28,31,32,33,34,35^ 4 randomized controlled trials,^16,19,23,36^ 1 mixed method,^29^ 1 cross sectional study,^26^ 1 prospective controlled assessment,^20^ and 1 cohort study.^30^ Research fields from the included studies were as follows: medicine^16,17,18,19,20,22,23,24,26,30,31,32,33,35^ (58.3%), occupational therapy^14,25,29^ (12.5%), physical therapy^14,28,29^ (12.5%), dentistry^13,34^ (8.3%), nursing^21,27^ (8.3%), speech therapy^15^ (4.2%), and athlete training^36^ (4.2%). The main objective of the included studies was to analyze the effectiveness of EBP taught to students in the health area. EBP teaching methods varied among studies as follows: time period varied from 4 hours workshop to 6 months period course; and format varied among seminars, workshops, courses, lectures, web-based learning modules, and conferences. Data were extracted through different types of pre- and post-intervention questionnaires or only post-intervention questionnaires. The majority of studies (n = 19, 79.2%), except for five,^16,19,21,23,26^ proved that teaching EBP was effective. A summary of the objectives, EBP teaching methods, how data were extracted, and conclusions based on the studies’ results is presented in **Table 3**. Due to the large heterogeneity, a meta-analysis of the results was not possible.

**Table 3:** Summary of objectives, EBP teaching method, how data was extracted, and conclusions based on studies’ results of all 24 included studies

Nº	(Author, year of publication) Research field	Objectives Sample Size (year of search)	EBP teaching method	How data was extracted	Conclusions based on studies’ results
1	(Arias, Peters, Broyles, 2017^13^) Dentistry	To develop a curriculum in biostatistics with various educational strategies to be applied in clinical practice. n = 3 (2016)	Needs assessment survey; curriculum design and implementation, with a series of 10 1-hour seminar sessions on specific learning outcomes; and curriculum evaluation, with knowledge tests and satisfaction survey.	28-item selected response extending matching and multiple choice knowledge test, focusing on improving their knowledge, high-order cognition, attitudes, and skills to develop their critical thinking when deciding the treatment.	The correct answer rate changed from 36.9% in the pretest to 79.8% in the post-test. Students showed high knowledge improvement in questions related to the identification of variable types and statistical test selection. They also benefited from the knowledge acquired in the didactic seminars and clinical learning activities allowing further critical analysis and discussions.
2	(Bennett, Hoffmann, Arkins, 2011^14^) Occupational therapy and Physical therapy	To investigate the improvement of health students’ attitudes, confidence and knowledge regarding EBP. n = 59 (2010)	Course of 13-week period, with 2 hours per week, using didactic lectures, tutorial and workshop formats, and a hands-on database searching session.	Pre- and post-course adapted questionnaire of the perceptions of occupational therapists toward EBP, and another regarding general practitioners’ self-rating of EBP skills.	The health students’ confidence regarding EBP skills, as well as their perceived and actual knowledge regarding EBP concepts were statistically significant after the course.
3	(Durieux, Maillart, Donneau, Pasleau, 2018^15^) Speech therapy	To evaluate the improvement of EBP competencies regards skills and knowledge. n = 104 (2018)	An educational and interactive module on EBP over 2 months.	Pre- and post-adapted Fresno test and a computer-based searching task requiring a search in PsycINFO.	The mean total score of the trained group was statistically significant in the pre and post-test, who made more progress in terms of EBP knowledge and skills.
4	(Feldstein, Maenner, Srisurichan, Roach, Vogelman, 2010^16^) Medicine	To analyze the improvement of residents’ EBP knowledge and better prepare them for effective clinical decision-making. n = 48 (2003 and 2004)	4-hour interactive EBP workshop for the treatment group (n = 23), as well as a journal club.	EBP knowledge and skills test were applied 6 and 18 months later on, with 25 multiple-choice questions.	There were no significant differences between treatment groups at either time point. No differences were detected in EBP knowledge between residents who did and did not participate in the workshop.
5	(George, Reis, Nothnagle, 2012^17^) Medicine	To describe the design, implementation, and evaluation of a curricular intervention tailored to individual residents. n = 26 (2008 to 2010)	A learning coach to develop EBP skills, with monthly half hour meetings devoted to EBP training.	Pre and post quantitative and qualitative methods, using a 10-item survey and 6 questions adapted from the Fresno Test of Competence in EBP.	The variable attitude was statistically significant in 4 of 10 items in the pre and post-test. The competence questionnaire was statistically significant in all items. Residents demonstrated improved knowledge and skills of EBP through the intervention.
6	(Green Ellis, 1997^18^) Medicine	To develop and implement an EBP curriculum and determine its effectiveness in improving residents’ EBP behaviors and skills. n = 34 (1995 to 1996)	7-week EBP curriculum based on adult learning theory, the educational strategy with resident directed tutorial format, use of real clinical encounters and specific EBP facilitating techniques for faculty (n = 19).	Pre and post questionnaires, with a survey of EBP behaviors, a survey of self-assessed EBP competence and an EBP skills test.	The case subjects significantly improved their scores on the EBP skills test. EBP curriculum improved residents’ EBM skills and certain EBM behaviors.
7	(Hadley et al., 2010^19^) Medicine	To evaluate the educational effectiveness of a clinically integrated EBP e-learning course among postgraduate medical trainees compared to a lecture-based course. n = 160 (2007)	One group received the e-learning EBM teaching program (n = 88) and the other received standard classroom-based standalone EBM teaching sessions of equivalent content (n = 72). The e-learning group were granted unlimited access for a period of 6 weeks to the e-learning materials via a project specific website.	Validated multiple-choice questions to assess EBM knowledge before accessing the e-learning materials and prior to the start of the teaching sessions. After completion of each module, the participants completed the same questions relevant to that module again.	There was no difference in the amount of improvement of knowledge between the two groups. The benefits should be considered a potentially cost-effective alternative to standard lecture-based sessions.
8	(Harewood Hendrick, 2010^20^) Medicine	To evaluate the impact of a workshop on the critical appraisal skills of medical trainees. n = 19 (2009)	6 hours EBP workshop, with three sessions of 2 hours each.	Nine research papers were emailed before the first lecture. Pre and post grading of the quality of these studies on a 3-point scale.	Total correct grading went from 39% before the course to 74% post course. There was an improvement in the participants’ knowledge of EBP skills and an improvement in their ability to critically assess published literature.
9	(Jackson, 2016^21^) Nursing	To determine the effectiveness of an EBP learning module within a nursing residency program. n = 29 (2014)	Learning module activities over 4 months.	Pre and post module EBP questionnaire, which consisted of 24 items with 7-point rating scales.	The variable practice was statistically significant in one of six items in the pre- and post-test. The variable attitude did not statistically differ in any item. The variable knowledge/skills was statistically significant in 4 of 14 items in the pre and post-test. Therefore, most items did not show statistical differences between the pre and post-test.
10	(Kenefick, Boykan, Chitkara, 2013^22^) Medicine	To evaluate the effectiveness of partnership between librarians trained with EBP and residents’ process of learning. n = 4 (2010)	Partnership between librarians trained with EBP and residents’ process of learning.	Pre and post-tests on how to form a clinical question, how to conduct searches and four cases to evaluate the residents’ baseline skills and efficiency.	After the alliance between the librarians and the residents, test scores went from 46.0% to 99.0%. Librarians and residents must be taught how to use available resources, especially the residents. Partnering with residents for the long term made toward better physician decision-making rewards the librarian, the physician, and ultimately the patient.
11	(Kortekaas et al., 2016^23^) Medicine	To report the results of a cluster randomized controlled trial among third year trainees comparing the effects of the integrated EBP training program with a stand-alone EBP training program. n = 79 (2011 to 2013)	EBP training in accordance with the five steps of the Sicily Statement. Main difference between the stand-alone (n = 40) and integrated EBP training program (n = 39) was the focus on the last two steps, which was adapted to emphasis the practical implication of research and to stress its clinical relevance.	EBP behavior, measured as guideline adherence and information-seeking behavior, as well as EBP attitude and knowledge.	Information-seeking behavior, guideline adherence, EBM attitude, and knowledge did not significantly differ between both groups.
12	(Ma, Chang, Krupat, 2021^24^) Medicine	To evaluate and quantify the benefits of an educational module on EBP. n = 111 (2021)	16 EBP 2 hours’ sessions on a weekly basis, with a computer laboratory and audiovisual aids.	Oral presentation on the standardized cases during the first two sessions and the last three sessions, with the utilization of EBP relevant electronic medical databases for selecting and organizing references of high-level evidence. Pre- and post-intervention questionnaires on EBP behavior or practice of applying relevant resources, as well as cognition or awareness of relevant resources.	The clinical scenario presentation had a statistically significant improvement comparing pre and post intervention. Self-reported changes on behavior and awareness presented statistically significant differences for post intervention.
13	(McCluskey Lovarini, 2005^25^) Occupational therapy	To measure the effect of a multifaceted intervention on EBP, on knowledge, skills, behaviors, and attitudes. n = 114 (2001 to 2003)	2-day workshop on EBP, with lectures, practical sessions and small group discussions.	Pre, post, and after 8 months written questionnaires of the adapted Fresno test of competence in EBP, divided into three sections.	Tests were statistically significant pre and post workshop, with 19.0% of participants scoring at least 50.0% of correct answers before the workshop, and after that 77.0%. However, changes in behavior were not maintained after 8 months, based on the frequency of searching and appraisal activities.
14	(Mlika, Ben Hassine, Charfi, Mezni, Jouini, 2019^26^) Medicine	To assess the acceptability of learning EBP by family doctors. n = 17 (2019)	Associated lecture sessions and teamwork participative methods.	A website was created for pre- and post-test.	There was no statistical difference in pre and post-tests.
15	(Moore, Watters, Wallston, 2019^27^) Nursing	To evaluate the impact of EBP courses on nursing students’ attitudes. n = 227 (2014)	6 months EBP course. An adapted EBP research instrument of the theory of planned behavior, including an EBP knowledge test.	An adapted EBP research instrument of the theory of planned behavior, including an EBP knowledge test.	The variables attitudes, self-efficacy, and behavior were statistically significant pre- and post-test for both groups (masters of science in nursing and doctorate of nursing practice). Advanced practice nurses are better prepared as leaders to implement EBP in the clinical setting, resulting in high quality of care and improved health outcomes.
16	(Perraton et al., 2017^28^) Physical therapy	To measure if there is a change in confidence and anxiety in knowledge of statistical terminology and concepts related to research design and EBP. n = 236 (2007 to 2014)	An intensive 3-week post-graduate course, which taught health research methods, biostatistics and EBP.	Pre and post questionnaires regarding confidence in methods and understanding of EBP.	The variables knowledge of statistical terminology and concepts related to research design and EBP were statistically significant pre- and post-tests. An intensive teaching program in health research methods and biostatistics and EBP was effective immediately post-course.
17	(Schweikhard, Hoberecht, Peterson, Randall, 2018^29^) Occupational therapy and Physical therapy	To evaluate the impact of the library tutorials on the information literacy skills of students in the EBP course. n = 449 (2012 to 2016)	The authors analyzed the impact of online library tutorials in association with the EBP course. Focuses were on students’ search strategies and cited sources pre and post the implementation of the tutorials.	Students wrote a “Step 5 Paper”, which was to synthesize each of the steps and reflect on the entire process.	When comparing the total number of studies cited in the pre- and post-tutorial Step 5 Paper, there was an improvement on the choice of papers’ quality. Before the tutorials, 73.0% of studies were of higher quality, after the tutorials that number increased to 81.0%.
18	(Soma, Homme, Jacobson, 2013^30^) Medicine	To analyze if tablet computers would improve EBP knowledge, skills, and behavior. n = 38 (2011 and 2012)	Two introductory sessions, review of EBM curriculum, team assignments for learning exercises, and orientation to tablet computers. A series of 45-minute laboratory sessions twice monthly, focused on speed and efficiency for real-time, clinical use of EBM.	Pre and post intervention tests: a written test of knowledge and an online survey of skills and behaviors, an adapted Pediatric based instrument for assessing resident education in EBP.	Pre-intervention test score showed median of 32.5 of 60 points, in contrast with 53.0 points post intervention. The survey revealed statistically significant improvement in four of seven EBP skills. EBP improved knowledge, skills, and behavior through the introduction of a tablet computer and laboratory sessions.
19	(Tavarez, Kenkre, Zuckerbraun, 2020^31^) Medicine	To determine if implementation of EBP curriculum has an effect on pediatric emergency medicine fellows’ scores on the relevant section of the in-training examination. n = 22 (2001 to 2017)	Two online, self-directed modules for fellows to access and review 3 to 4 weeks before their assigned session. Approximately 2 to 3 weeks before the fellows’ scheduled session, they also had 1 hour session coaching and feedback. Monthly EBP sessions, with critical appraisal and discussion of 1 article.	Post sessions, an EBM worksheet was distributed to all fellows, with three to five questions about the chosen article. They obtained raw sub-scores on the scholarly activities section and raw sub-scores for the Emergencies Treated Medically section of the in-training examination questions.	The multivariate modeling demonstrated a higher performance of the students after the implementation of the EBP curriculum. Pediatric emergency medicine educators could consider using fellows’ scores on this section of the in-training examination to assess the effect of their EBP curriculum.
20	(Thom, Haugen, Sommers, Lovett, 2004^32^) Medicine	To evaluate the EBP curriculum based on an individual block rotation and the EBP skills throughout the residency program. n = 13 (2001 to 2003)	2-week EBP rotation.	A written test of their EBP skills and knowledge before and immediately following the rotation.	The use of PubMed/Medline, other Web-based EBP resources, EBP tools and principles (critical appraisal), as well as their confidence over the course of the rotation statistically increased. Residents and faculty felt that the answers provided by the EBP intern provided useful information and led to changes in patient care within the residency program.
21	(Thomas et al., 2005^33^) Medicine	To determine if an EBP conference program would enhance resident competency at EBP or if resource intensive, small group discussions would be required. n = 46 (1997 to 1999)	EBP conferences and small-group discussions were integrated into an internal medicine curriculum to compare the efficacy of EBP competency. The students were divided into two groups and they were compared with one another.	Written examination comparing both groups.	Small-group discussion participants scored higher when compared with conference participants, also with increased confidence and high satisfaction. Therefore, small-group discussions resulted in increased EBP knowledge, increased confidence with critical appraisal skills, and high satisfaction compared with a conference-based format.
22	(Wadgave, Khairnar, Kadu, Chadha, Wadgave, 2020^34^) Dentistry	To determine the effectiveness of a formal education workshop on EBP to undergraduate dental students. n = 50 (2019)	Four interactive seminars, practical sessions, and home assignments held for 2 days at the EBP workshop.	EBP knowledge, attitudes, access and confidence questionnaire before and after the course.	EBP knowledge was statistically significant in 6 of 10 items in the pre- and post-test. Skills in accessing evidence were statistically significant in four of nine items in the pre- and post-test. A significant improvement was noted in positive attitudes of students toward EBP. Participants gained moderate confidence in critical appraisal skills.
23	(Wang et al., 2017^35^) Medicine	To assess the effectiveness of an EBP course on the Chinese medical students’ critical thinking skills and dispositions. n = 158 (2017)	EBP course with 32 hours of Clinical Epidemiology and 24 hours of EBP Approaches, each taught for 8 consecutive weeks in a sequence.	A survey based on the Chinese version of the Critical Thinking Disposition Inventory before and after the course.	The results showed a significant improvement in confidence and progress in inquisitiveness after they completed an EBP course.
24	(Welch, Van Lunen, Hankemeier, 2014^36^) Athlete training	To assess whether an EBP educational intervention enhanced knowledge of EBP concepts among athletic trainers. n = 175 (2011)	10 web-based learning modules, where the experimental group had access to the web-based modules for 4 weeks, whereas the control group had no direct responsibilities for the investigation.	Knowledge assessment consisted of 60 multiple-choice questions pertaining to concepts presented in the 10 modules.	The experimental group obtained higher scores on the post-assessment than the pre-assessment. No differences were identified among time instances within the control group. Therefore, the educational intervention web-based modules was an effective mechanism to increase knowledge of foundational EBP concepts.

EBP = evidence-based practice; EBM = evidence-based medicine.

## DISCUSSION

This systematic review aimed to explore the effectiveness of EBP in higher education. The results showed that 79.2% of the studies proved EBP effectiveness.^13,14,15,17,18,20,22,24,25,27,28,29,30,31,32,33,34,35,36^ This study’s secondary objective was to understand the methods used to teach EPB, which varied over time (from 4 hours to 6 months), format (seminars, workshops, courses, lectures, web-based learning modules, and conferences), and types of pre-and/or post-questionnaires.

A systematic review published in 2016^10^ suggested that technology focused mainly on teaching the critical appraisal of information could influence abilities, knowledge, and attitudes regarding the use of EBP. Our results determined that the development of such abilities for EBP implementation depended on the construction and revision of EBP curriculum, through working on the teaching/learning time period, effective from at least 6 hours workshop, once Feldstein^16^ did not show effectiveness with their 4 hour EBP workshop; format of EBP teaching, which could vary a lot, as long as it had enough time to cover main EBP points; and type of evaluation to check if students were really able to implement EBP, once assessment strategies were also essential.^37^ The majority of higher education universities offers biostatistics discipline in their curriculum; however, such discipline rarely involves EBP in its practice components.^13,28^ Our recommendation is focused on the development of a new EBP curriculum or even a restructuration of the biostatistics curriculum. The main proposal is to encourage students to engage in reflective thinking, with active methods of searching for knowledge, critical appraisal skills, behavior, and attitudes toward reading scientific articles.^16,17,18,20^ Other suggestions for the development of the EBP curriculum have been listed elsewhere.^38^

This study has some limitations in terms of our descriptive results, as it was not possible to center the data in a meta-analysis to obtain more accurate results. Further research could analyze the association of EBP teaching effectiveness with different variables, such as teaching period and format. The strength of this review lies in the methodological analysis of the included studies.

## CONCLUSION

In conclusion, most of the studies included in the present review demonstrated the effectiveness of the chosen EBP teaching method; however, future research should explore the factors that could be associated with the improvement of such effectiveness. This study used educational tools through EBP teaching methods. Additionally, it is important for health professionals to use EBPs to ensure more effective treatment of patients.
